# 2017 Update on Ovarian Cancer Peritoneal Carcinomatosis Multimodal-Treatment Considerations

**DOI:** 10.1155/2018/5284814

**Published:** 2018-04-05

**Authors:** Evgenia Halkia, George Chrelias, Charalambos Chrelias, Jesus Esquivel

**Affiliations:** ^1^3rd Gynecologic Clinic, University Teaching Hospital “Attikon”, National and Kapodistrian University of Athens Medical School, Athens, Greece; ^2^American Society of Peritoneal Surface Malignancies (ASPSM), Clarkesville, MD, USA; ^3^General and Oncology Surgery Clinic, Frederick Memorial Hospital, Frederick, MD, USA

## Abstract

Ovarian cancer peritoneal carcinomatosis requires a multimodal-treatment approach. Current treatment considerations are analyzed in this update and include the management of recurrent malignant ascites and the understanding of its pathophysiology, the role of peritoneal washing cytology in detecting peritoneal metastases, capsular invasion and ovarian cancer histologic type, interpretation of pretreatment Ca-125 levels at different time points of ovarian cancer therapeutic management, characteristics of 10-year survivors of high-grade ovarian cancer, and the role of lymphadenectomy in ovarian cancer peritoneal carcinomatosis. This update also includes current considerations on the role of cytoreductive surgery and hyperthermic intraperitoneal chemotherapy in ovarian cancer peritoneal carcinomatosis as well as relevant ongoing phase III randomized controlled trial protocols.

## 1. Introduction

The aim of this update is to bring to light current trends and considerations in the multimodal management of ovarian cancer.

### 1.1. Diagnostic and Therapeutic Implications in the Treatment of Ovarian Cancer Malignant Ascites

Malignant ascites has been known as one of the major factors to negatively affect quality of life and prognosis in epithelial ovarian cancer (EOC) patients. It represents a tumor-friendly microenvironment with cellular (tumor cells and stromal cells) and acellular (soluble factors) components [1].

Cellular components such as adipocytes, fibroblasts, endothelial and mesothelial cells, and adipocyte tissue- and bone marrow-derived stem cells may show phenomena such as activation of angiogenesis and growth as well as interactions of ovarian cancer cells and peritoneal mesothelial cells. All of these interactions are very important for the tumor growth [2–4].

Tumor cells in ascites may play a role in recurrence. Such tumor cells can form aggregates with nonadherent properties named “spheroids” [5]. It has been proposed that spheroids are responsible for the invasive and metastatic properties of EOC tumor through their transition to a motile type, responsible for invasiveness and disease recurrence [1, 6].

“Exosomes” comprise nanosized particles excreted by cellular components of the ascitic fluid, with the potential to influence EOC progression through tumorigenic factors [7]. Disease-specific biomarkers such as miR-200c, miR-214, CA-125, Muc-1, and CD24 are contained in exosomes and may alter gene expression in cells [1, 7].

EOC cells express a great heterogeneity in metabolism, gene expression, and metastatic potential. This heterogeneity responds to both genetic and environmental contributing factors. During disease progression, oncogenic tumor-suppressive signals from cellular and acellular components in the ascitic fluid change its consistency continually [8, 9]. It has been indicated that ascites may provide a microenvironment that is tumor protective against chemotherapy and apoptosis [10, 11].

Ascites might be present either in benign or malignant EOC tumors. Abdominocentesis for cytological differential diagnosis has high specificity but low sensitivity and might require multiple interventions resulting in patient discomfort and inaccuracy [12].

Different kinds of tumor markers such as VEGF (vascular endothelial growth factor) matrix metalloproteinase are of minor diagnostic value as different types of ovarian tumors produce a different ascitic microenvironment. However, the consistency of the ascitic fluid may give significant information, on a micromolecular level, about the efficacy of targeted agents for treatment such as “bevacizumab” [13].

Fossati et al. reported the immunological changes in the ascites of cancer patients after intraperitoneal administration of the specific antibody “catumaxomab” (anti-EpCAMXanti-CD3), with good outcomes [14].

Because of the increased absorption/production of the lymph and the alteration of the capillary permeability, ovarian cancer ascites can make the patient feel uncomfortable, painful, anorexic, dyspneic, and distended [15].

Because of the diversity of the underlying pathophysiology, EOC malignant ascites preclude therapeutic manipulations, that is, diuretics and so forth, to alleviate symptoms [16, 17].

Other treatment choices such as peritoneal-venous shunts, radiolabeled antibodies, and biologic agents have not been established as standard of care so far [18–20].

Palliative laparoscopic HIPEC (hyperthermic intraperitoneal chemotherapy) has been explored to treat debilitating recurrent malignant ascites [21, 22]. A multi-institutional retrospective analysis of 52 patients with refractory malignant ascites by Valle et al. reported one clinical recurrence of the ascites after laparoscopic HIPEC and an important improvement in performance status postoperatively. Abdominal sclerosis and induction of dense adhesions, rather than the direct cytotoxic effect of the IP (intraperitoneal) drug, were the major factors of efficacy of this technique [23]. In another phase I study by Ozols et al., the authors reported sclerosing peritonitis and subsequent pain as the dose limiting factors for intracavitary chemotherapy with doxorubicin in patients with advanced EOC [24, 25].

### 1.2. The Role of Peritoneal Washing Cytology in Ovarian Malignancy

In a recent retrospective study by Naz et al., a total of 60 cases of women with ovarian tumors who underwent TAH (total abdominal hysterectomy) with BSO (bilateral salpingo-oophorectomy) and omental/lymph node sampling [26] were included. Any free abdominal fluid was aspirated at the time of surgery. In the absence of free fluid, peritoneal washing was done with 50–100 ml of N/S. Correlation of peritoneal cytology with various histologic parameters was performed. Out of the 60 cases, 56 were surface epithelial tumors, 2 were germ cell tumors, and 2 were metastatic carcinomas. Capsular invasion was seen in 61% of the cases and omental metastasis in 51% of the cases. Serous carcinoma was found to have significantly higher frequency of positive peritoneal cytology (76.9%) compared to endometrioid and mucinous carcinomas (44% and 25%, resp.). A significant positive correlation was seen between positive peritoneal cytology and capsular invasion and omental metastases with a *p* value of <0.001. The authors concluded that in addition to being an indicator of peritoneal metastasis, positive cytology also correlates with capsular invasion and histologic type in ovarian cancer tumors. Therefore, it should always be used as an adjunctive tool in the surgical management of ovarian malignancies [26].

### 1.3. Interpretation of CA-125 Pretreatment Levels in Ovarian Cancer

A retrospective study by Morales-Vásquez et al. reported 1009 patients with EOC and the association of CA-125 measurements before any chemotherapy or surgical cytoreduction, with the clinical stage, histology, differentiation, grade, and survival rate of these patients [27]. The abnormal level (>35 U/ml) of CA-125 was observed in 99% of serous carcinomas. Abnormal CA-125 values were observed in 89% of the endometrioid subtype and 69% of the mucinous tumors, with the highest absolute value of CA-125 observed in serous carcinomas of the ovary, surpassing any other histological subtype. Clinical stages III and IV displayed increased CA-125 values compared to stages I and II. Undifferentiated carcinomas showed the highest level of CA-125 compared to the moderately differentiated grade. Surprisingly, survival evaluation by Kaplan-Meier analysis including only high-grade serous carcinoma at FIGO stages III and up (*n* = 57) demonstrated 57.1% chances of survival in patients with CA-125 pretreatment levels higher than 500 U/ml. Survival was 26.7% in patients with CA-125 pretreatment levels lower than 500 U/ml, and the hazard ratio for CA-125 values less than or equal to 500 U/ml was 2.28, 95% CI 1.08–4.84, *p* = 0.032. The authors concluded that (a) values of CA-125 higher than 500 U/ml in high-grade serous carcinoma with FIGO stage III or higher resulted in an enhanced survival rate of these patients, and (b) probably, patients with a carcinoma generating high levels of CA-125 react differently to disease or to treatment, resulting in an increased survival rate; these findings support the theory that EOC is not a single entity, being at least five diseases with different natural histories [27].

Another study conducted by Zeng et al. examined 118 patients with advanced EOC, primary carcinoma of the fallopian tube, and peritoneal carcinoma to determine whether reduction of CA-125 levels is a predictive factor for cytoreduction to no visible residual disease (NVRD) and chemotherapeutic sensitivity [28]. This was a single team-based study and patients included were treated with NAC-IDS (neoadjuvant chemotherapy-interval debulking surgery) by one gynecologic oncologist. 37 patients (31.4%) underwent resection to NVRD. The median serum CA-125 level at presentation and before IDS was 1814.5 U/ml and 205.9 U/ml, respectively. In the univariate analysis histology, a preoperative CA-125 level of less than or equal to 200 U/ml and a >80% reduction of CA-125 levels between presentation and IDS were significantly associated with the likelihood of NVRD (*p* = 0.014, 0.000, 0.000, resp.). Multivariate analysis showed that a preoperative CA-125 level of equal or less than 200 U/ml was the only independent predictor of NVRD (odds ratio 3.667, 95% CI 1.337–10.057, *p* = 0.012). Preoperative CA-125 levels of less than or equal to 200 U/ml was also significantly associated with chemotherapy-sensitive disease in the univariate analysis (*p* = 0.037). The authors concluded that the percentage change and the absolute level of CA-125 after the first cycle of NAC were not associated with “optimal cytoreduction” suggesting that evaluation of CA-125 levels after a single cycle may have little predictive value. However, they found that a preoperative CA-125 level of less than or equal to 200 U/ml was an independent predictor of optimal cytoreduction to NVRD [28].

### 1.4. Characteristics of 10-Year Survivors with High-Grade Serous Ovarian Carcinoma (HGSOC)

A multicenter research consortium was established between five participating academic centers. 203 patients were included in this study by Dao et al., and the clinical features in women surviving HGSOC for 10 or more years were identified [29]. The majority of patients had stage IIIc (72.4%) disease at presentation. Of those who underwent primary cytoreductive surgery, optimal cytoreduction was achieved in 143 (85.6%) patients. After a median follow-up of 144 months, 88 (46.8%) of patients did not develop recurrent disease after initial treatment. Unexpected findings from this survey were as follows: 14% of patients had suboptimal cytoreduction, 11% of patients had an initial platinum-free interval of <12 months, and nearly 53% of patients had recurrent disease, yet still surviving more than ten years after diagnosis. Long-term survivors of HGSOC generally had optimal cytoreduction and primary platinum-sensitive disease. The majority of patients developed recurrent disease; however, many remained disease-free for more than ten years. The authors concluded that long-term survivors exist both with and without multiple recurrences. They appear to have a slightly younger age than the average patient with advanced-stage HGSOC and are likely to have had optimal cytoreduction. They indicated that there are intrinsic biologic factors associated with platinum sensitivity that are generally associated with long-term survival, but surprisingly, a small fraction of patients who had either suboptimal cytoreduction or primary platinum resistance achieved long-term survival. Of importance was the indication that the low use of neoadjuvant chemotherapy may be directly associated with long-term survival [29].

### 1.5. Lymphadenectomy in Ovarian Neoplasms (LION) Study

While some previous research has suggested a survival advantage to lymphadenectomy, there is no level 1 evidence regarding the role of systematic pelvic and para-aortic lymphadenectomy (LNE) in patients with advanced ovarian cancer (AOC) with macroscopic complete resection and clinically negative lymph nodes (LN). Therefore, surgical management regarding LNE worldwide is very heterogeneous [30]. The LION study, a phase III RCT conducted by Harter et al., examined 657 patients with newly diagnosed stage IIB–IV ovarian cancer who had undergone macroscopic complete resection and had pre- and intraoperatively negative lymph nodes. They were randomly assigned to lymphadenectomy (*n* = 323) or control (*n* = 324). The results of the study were presented at the American Society of Clinical Oncology (ASCO) 2017 annual meeting. The median number of removed lymph nodes in patients randomized to LNE was 57 (pelvic 35 and para-aortic 22). Post-op platinum/taxane-based chemotherapy was applied in 85% of the patients in the no-LNE arm and 80% in the LNE arm. Microscopic metastases were diagnosed in 56% of the patients in the LNE arm. Median OS (overall survival) was 69 months in the no-LNE arm and 66 months in the LNE arm (HR 1.06, 95% CI 0.83–1.34, *p* = 0.65), and the median PFS (progression-free survival) was 26 months in both arms (HR 1.11, 95% CI 0.92–1.34, *p* = 0.30). Surgery in the LNE arm was 64 minutes longer (means: 352 versus 288 min), resulting in a higher median blood loss (650 versus 500 ml) and higher transfusion rate (67% versus 59%). Serious postoperative complications occurred more frequently in the LNE arm (e.g., rate of relaparotomies 12.1% versus 5.9% (*p* = 0.006), hospital readmittance rate 8.0% versus 3.1% (*p* = 0.006), and deaths within 60 days after surgery 3.1% versus 0.9% (*p* = 0.049)). The authors concluded that systematic pelvic and para-aortic LNE in patients with AOC with both intra-abdominal complete resection and clinically negative LN improve neither overall nor progression-free survival despite detecting (and removing) subclinical retroperitoneal lymph node metastases in 56% of the patients [30].

### 1.6. Update on the Role of HIPEC (Hyperthermic Intraperitoneal Chemotherapy) and CRS (Cytoreductive Surgery) in Ovarian Cancer

A systematic review and meta-analysis by Huo et al. studied a total of 9 comparative studies and 28 studies examining HIPEC + CRS for primary and/or recurrent EOC [31]. Meta-analysis of the comparative studies showed that HIPEC + CRS + chemotherapy had significantly better 1-year survival compared with CRS + chemotherapy alone (OR: 3.76, 95% CI 1.81–7.82). The benefit of HIPEC + CRS continued for a 2-, 3-, 4-, 5-, and 8-year survival compared to CRS alone (OR: 2.76, 95% CI 1.71–4.26; OR: 5.04, 95% CI 3.24–7.85; OR: 3.51, 95% CI 2.00–6.17; OR: 3.46 95% CI 2.19–5.48; and OR: 2.42, 95% CI 1.38–4.24, resp.). Morbidity and mortality rates were similar. Pooled analysis of all studies showed that among patients with primary EOC, the median 1-, 3-, and 5-year OS rates were 88.2%, 62.7%, and 51% (46.1 months). For recurrent EOC, the median 1-, 3-, and 5-year overall survival rates were 88.6%, 64.8%, and 46.3% (34.9 months). A step-wise positive correlation between completeness of cytoreduction and survival was found. The authors concluded that the addition of HIPEC to CRS and chemotherapy improves OS rates for both primary and recurrent EOC [31].

Another multicenter study by Di Giorgio et al. investigated 511 patients with advanced ovarian cancer who underwent CRS + HIPEC and analyzed data at eight treatment time points: primary debulking surgery (PDS); interval debulking surgery after partial response, after no response, and after a pathologic complete response to neoadjuvant chemotherapy; first recurrence with a progression-free interval 12 months or 12 months in patients who underwent further chemotherapy before CRS and HIPEC; and patients who underwent two or more CRS procedures and chemotherapy lines before CRS and HIPEC [32]. At a median follow-up of 53.8 months, OS was 54.2 months (95% CI 44–58.4) and PFS was 16.6 months (95% CI 14.7–19.1). Outcome analysis in patients in whom CRS + HIPEC was used for primary advanced cancer or recurrent ovarian cancer showed significant differences in OS and PFS according to the time points analyzed. Multivariate analysis identified completeness of CRS, peritoneal cancer index, and the times when the patients underwent CRS + HIPEC as independent prognostic factors. This multicenter study sheds light on how to simplify the process of selecting patients with advanced ovarian cancer who need to undergo HIPEC + CRS, specifying when this integrated procedure might have the greatest outcome benefit [32].

An ongoing phase III RCT (randomized controlled trial), named “CHORINE” by Ansaloni et al., compares two-year disease-free survival of CRS (cytoreductive surgery) and HIPEC (HIPEC, CDDP(cisplatin) + paclitaxel) versus CRS alone in stage IIIc unresectable epithelial tubal/ovarian cancer with a partial or complete response after 3 cycles of first-line chemotherapy (CBDCA + paclitaxel). Results are pending [33].

“CHIPOR” is another ongoing phase III RCT by Classe et al. “CHIPOR” hypothesizes that the adjunction of platinum HIPEC in first-relapsed EOC is able to improve the median OS (overall survival) by 12 months. The patients included in the study receive, before surgery, a second-line chemotherapy—a platinum-based regimen with either carboplatin-paclitaxel or carboplatin-caelyx. At the end of six courses of IV chemotherapy, if the patient is a responder, and if complete CRS is possible, then the patient will be operated 5 to 6 weeks after the second-line chemotherapy cycle. During surgery, the patient is randomized (if complete CRS is done or not) to either (a) treatment A: maximal CRS without HIPEC or (b) treatment B: maximal CRS with HIPEC. Results are pending [34].

“Hipecova” is an ongoing phase III RCT by Campos et al., evaluating the efficacy of HIPEC with paclitaxel in advanced ovarian cancer. There are two arms: (a) the HIPEC arm: CRS + HIPEC with paclitaxel (175 mg/m^2^) × 60 min at 42–43°C followed by postoperative systemic IV chemotherapy with carboplatin + paclitaxel × 6 cycles and (b) the no HIPEC arm: CRS followed by postoperative systemic IV chemotherapy with carboplatin + paclitaxel. Results are pending [35].

A phase III multicenter prospective RCT by Cui et al. examines the safety and efficacy of HIPEC as NACT and postoperative chemotherapy after IDS in the treatment of advanced-stage EOC. Patients in arm A will have (1) HIPEC with paclitaxel (100 mg/m^2^), paclitaxel (75 mg/m^2^) + cisplatin (75 mg/m^2^) intraperitoneally in succession; (2) 2 cycles of NACT: paclitaxel 175 mg/m^2^ IV > 3 hr + carboplatin IV > 1 hr every 3 weeks; (3) IDS; (4) HIPEC with paclitaxel 100 mg/m^2^, paclitaxel (75 mg/m^2^) + cisplatin (75 mg/m^2^) intraperitoneally in succession; and (5) 2 cycles of ACT (adjuvant chemotherapy): paclitaxel 175 mg/m^2^ IV > 3 hr + carboplatin IV > 1 hr every 3 weeks. Patients in arm B will have (1) 3 cycles of NACT: paclitaxel 175 mg/m^2^ IV >3 hr + carboplatin IV > 1 hr every 3 weeks; (2) IDS; and (3) 3 cycles of ACT: paclitaxel 175 mg/m^2^ IV > 3 hr + carboplatin IV > 1 hr every 3 weeks. This study has not opened yet for recruitment [36].

Another phase III RCT study by van Driel et al. evaluates the safety and efficacy of the addition of HIPEC to secondary debulking surgery in stage III ovarian cancer. The study is not recruiting anymore. Results are pending [37].

## 2. Conclusions

There is a need for a more detailed understanding of the role of ascites-regulated molecules on subsets of ovarian cancer cells, through further studies that will highlight both genetic and responsive heterogeneity as well as chemoresistance mechanisms in ovarian cancer malignant ascites.

Peritoneal washing cytology has been proven to be an indicator of peritoneal metastases, capsular invasion, and histologic type in ovarian tumors and has been implemented in ovarian cancer guidelines as an adjunctive diagnostic tool.

Laparoscopic HIPEC is an emerging tool in the diagnosis, staging, and treatment of ovarian cancer peritoneal carcinomatosis patients. Because of the limited amount of data, cautious approach is recommended, and all patients should be part of an investigational protocol.

Pretreatment levels of CA-125 (>500 U/ml) in FIGO stage III OC patients seem to promote an enhanced survival rate. However, further studies need to be implemented on the subject.

Lowering CA-125 levels preoperatively to <200 U/ml may predict cytoreduction to no visible disease in patients with EOC, primary carcinoma of the fallopian tube, and peritoneal carcinoma.

Long-term survivors (10 years or more) with HGSOC may have favorable clinical features such as a history of optimal surgical cytoreduction and platinum-sensitive disease as well as recurrent disease. These results need to be confirmed through phase III RCTs.

The “LION” study, a large phase III RCT, examined lymphadenectomy in ovarian neoplasms. The results of the study showed that patients with advanced ovarian cancer who undergo a complete resection need not also undergo systematic lymphadenectomy because it has no effect on PFS or OS.

A recent systematic review and meta-analysis as well as a retrospective multicenter study suggested that HIPEC + CRS shows a survival benefit over CRS alone in EOC patients. Results from ongoing phase III RCTs on the efficacy of HIPEC and CRS are pending.

## 3. Therapeutic Considerations in the Selection of Treatment Approach of Ovarian Cancer Peritoneal Carcinomatosis

- Malignant ascites in OC (ovarian cancer) peritoneal carcinomatosis negatively affects quality of life either at primary diagnoses or at recurrence. It presents a challenge for the gynecologic oncologist as well as the medical oncologist as it indicates disease progression and worse prognosis and it may become refractive to treatment. Many times, end-stage OC patients suffer not so much of tumor progression, but of visceral organ obstruction, because ascites, even if evacuated, creates pseudomembranes and adhesions in the abdominal cavity that are refractory to any treatment approach. Understanding the pathophysiology underlying the generation and absorption of ascitic fluid is the cornerstone in the treatment plan of patients, as OC peritoneal carcinomatosis has different stages of disease progression. Laparoscopic HIPEC, as a minimally invasive treatment approach, especially as a palliation for refractory ascitic accumulation, needs to be seriously taken into consideration and examined in phase III RCTs.
- The standard clinical use of the tumor marker CA-125 has been examined in many studies with emphasis being given to the levels of the marker in comparison to that before treatment. The exact role and contribution of CA-125 in disease progress, recurrence, and response to treatment is still being followed and examined in many recent studies.
- A significant positive correlation has been established in many studies, between a positive peritoneal washing cytology and a capsular invasion in EOC peritoneal carcinomatosis, which needs to be included as an adjunctive diagnostic method in the treatment approach of the disease.
- The ten-year survivorship of patients with advanced-stage EOC has been associated with a younger age at diagnosis, a history of optimal cytoreduction, platinum sensitivity, and a low use of neoadjuvant chemotherapy treatment.
- Although systemic lymphadenectomy in advanced-stage EOC was advised in a previous research, a phase III RCT named “LION” concluded that systemic lymphadenectomy does not provide neither any progression-free interval nor any overall survival benefit for these patients.
- Finally, the clinical significance of the application of HIPEC during the different stages of EOC disease progression is gaining more and more acceptance in the gynecologic oncology treatment algorithm, and hopefully it will be soon included in the international guidelines as a standard of care for selected patients that will benefit.
